# Practical Departments

**Published:** 1894-11-24

**Authors:** 


### PRACTICAL DEPARTMENTS.

### THE LAUNDRY EXHIBITION.

As cleanliness is to health so is laundry work to cleanliness, and thus the laundry exhibition, which for the past week has been open at the Agricultural Hall, Islington, is of considerable interest to sanitarians and more especially to hospital authorities.

The economic and hygienic advantages of such modern appliances as have been there shown for the efficient carrying out of laundry work on an extensive scale are now too widely recognised to need much discanting upon here, more especially as in recent articles on the subject we have already discussed them.

Of late years improvements have followed with almost bewildering rapidity one upon the other in the machinery in use in large laundries. Considering the systems advocated by the various manufacturers, as shown *en masse* at the recent exhibition, we find that the differences between them are mostly in details, the general principles followed being more or less the same.

An initial difficulty to be overcome in washing clothes on a large scale is the hardness of the water which has to be used. Various ingenious methods of softening water for this purpose have been brought forward, and occupied a prominent position at the exhibition, notably Andrew Howatson's patent water softener, shown by Messrs. Thwaites Bros. (Limited), of Bradford. Others were exhibited by Messrs. Stack and Brownlow, Straker, Whitworth, and Co., and by Doulton and Co. Of rotary washing machines were to be seen an infinite variety, many of the ordinary type now in use in most laundries, and some showing special features, such as the division of the inside cylinder into four or six compartments.

Thomas Broadbent and Sons, of Huddersfield, show what they call the "Torrent Washer," in which a sort of water-wheel is fixed on the outside of the rotating barrel, so as to carry water up to the top and discharge it in a torrent upon the clothes as they fall, claiming that thereby they not only wash the clothes better but prevent what the laundry people call "roping." We were especially struck by the excellence of the washers for hospital and institutional use shown by Messrs. Clements, Jeakes, and Co., adapted for work of the heaviest nature, as well as for finer washing. Experience shows that money spent on procuring the best and strongest machinery when fitting up laundries for hospitals, &c., is wisely laid out, and is in the end a source of economy. This point was brought out by a fact elicited from Messrs. Clements and Jeakes' representative at the exhibition in connection with a large steam-heated ironing machine also shown by this firm. Some years ago in fitting up the laundry department of a new Asylums Board hospital it was suggested that one of these ironing machines should be put in, the authorities objecting to the proposal on the score of the greater expense as compared with the older system. When, however, lately the question again came up in connection with a recently-erected hospital, the Board put in not only one but two of these machines. Of "hydro-extractors" there is no end. Prominent among them was one shown by Mr. R. G. Whittaker, which was made to attract the attention of the crowd by the syren-like shriek which it produced when running empty at a high speed. Of course this does not occur when loaded. One well-known hydro-extractor, shown by Thomas Broadbent, differs from the rest in two ways that should be noted; first in being suspended in such a way as to prevent the transmission of vibration to the foundations, and second in being run by a small steam cylinder and piston attached to the framework, so that it is entirely free from straps, and can be run independently of any engine. Both these points seem of some importance in regard to hospital laundries.

It is interesting to note how completely the older plan of mangling has been superseded by the modern system of such ironers as the one we have already mentioned. The clothes are taken straight from the centrifugal wringing machines or hydro-extractors, and put through the ironers, these latter consisting of a series of heated steel rollers, covered usually with felt. An enormous quantity of work can be done in this way. As showing how completely these machines are taking the place of the "box-mangle," at present generally to be found in hospital laundries, these latter were hardly represented in the exhibition. A complete revolution, too, seems likely to take place in the process of drying. Till quite recently, and in fact at the present time, the common plan has been to place the clothes upon "horses," which are run into a closet heated with hot water pipes, and more or less efficiently ventilated. The newer plan is to use a room through which a blast of hot air can be passed at a considerable velocity. The clothes are hung in the room, the door is shut, and the heated air, propelled by a fan, is driven in large volume through the room and rapidly carries off all moisture. In regard to economy, especially of time, the new process is statedto be much better than the old, and as to sanitary value there can be no doubt that a proceeding like this, in which the clothes are freely waved about in a strong current of dry air, must be far superior to the old baking process in the drying closet. The Blackman Ventilating Company and Glover and Hobson exhibit these hot draught drying-rooms, the former showing how by moving a shutter the hot air can be swept out by a current of cold when the drying is completed, so as to save the workpeople the risk of entering the hot room, and the latter exhibiting a system by which the heat required may be obtained from the exhaust steam of the engine; in fact, they claim that by this arrangement, where an engine is used for laundry work, the whole of the heat required for drying can be obtained from what otherwise would be a waste product. Under these circumstances it is reasonable to conclude that before long the method of drying by heated closets will have given place to this newer and more hygienic system.

